# Hip Joint Capsular Anatomy, Mechanics, and Surgical Management

**DOI:** 10.2106/JBJS.19.00346

**Published:** 2019-09-20

**Authors:** K.C. Geoffrey Ng, Jonathan R.T. Jeffers, Paul E. Beaulé

**Affiliations:** 1 MSk Lab, Department of Surgery and Cancer, Imperial College London, London, United Kingdom; 2 Department of Mechanical Engineering, Imperial College London, London, United Kingdom; 3 Division of Orthopaedic Surgery, University of Ottawa, Ottawa, Ontario, Canada

## Abstract

➤ Hip joint capsular ligaments (iliofemoral, ischiofemoral, and pubofemoral) play a predominant role in functional mobility and joint stability.
➤ The zona orbicularis resists joint distraction (during neutral positions), and its aperture mechanism stabilizes the hip from adverse edge-loading (during extreme hip flexion-extension).
➤ To preserve joint function and stability, it is important to minimize capsulotomy size and avoid disrupting the zona orbicularis, preserve the femoral head size and neck length, and only repair when or as necessary without altering capsular tensions.
➤ It is not fully understood what the role of capsular tightness is in patients who have cam femoroacetabular impingement and if partial capsular release could be beneficial and/or therapeutic.
➤ During arthroplasty surgery, a femoral head implant that is nearly equivalent to the native head size with an optimal neck-length offset can optimize capsular tension and decrease dislocation risk where an intact posterior hip capsule plays a critical role in maintaining hip stability.

Hip joint capsular ligaments serve a fundamental role in balancing functional mobility and joint stability. Although the anatomy of hip capsular ligaments has been well described in the literature^1-4^, the knowledge of its characteristics and contributions toward hip mechanics and disease processes are evolving. More importantly, how the hip capsule is managed during surgical interventions (preservation and arthroplasty) and its effects on joint function are increasingly recognized^5-8^. Several recent laboratory studies have provided new insights into functional mobility and stability of the hip joint that should be carefully considered during surgery^9-20^. The purpose of this review was to provide a comprehensive summary of the functional anatomy and material characteristics of the hip capsule and the effects of different capsular management techniques during arthroscopic and arthroplasty procedures on joint function.

## Characteristics

### Anatomy

**Fig. 1:**
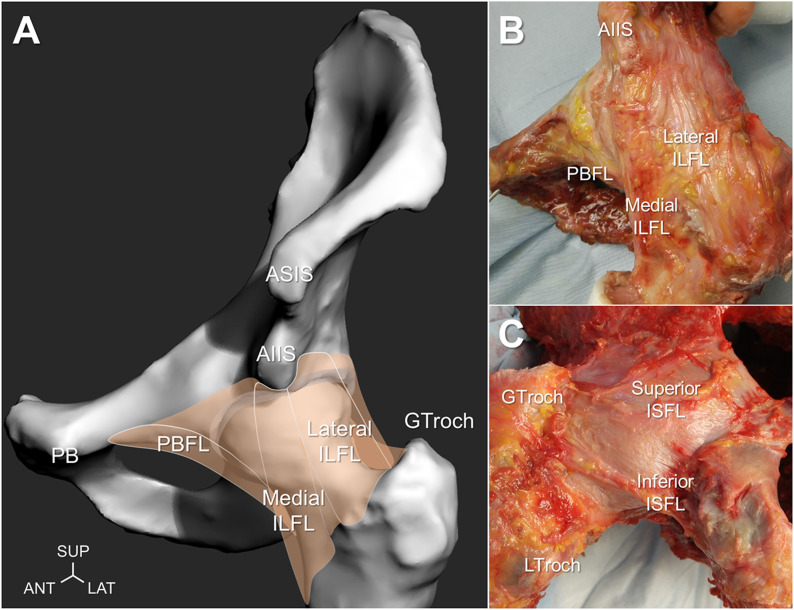
Anatomy of the capsular ligaments illustrated with a left-sided hip model in neutral position (**Fig. 1-A**) and showing the anterior view of a cadaveric hip specimen in external rotation (**Fig. 1-B**) and the posterior view of a cadaveric hip specimen in internal rotation (**Fig. 1-C**). The lateral and medial branches of the iliofemoral ligament (ILFL), pubofemoral ligament (PBFL), superior and inferior fibers of the ischiofemoral ligament (ISFL), anterior superior iliac spine (ASIS), anterior inferior iliac spine (AIIS), pubis (PB), and greater and lesser trochanters (GTroch and LTroch) are indicated. SUP = superior, ANT = anterior, and LAT = lateral.

**Fig. 2:**
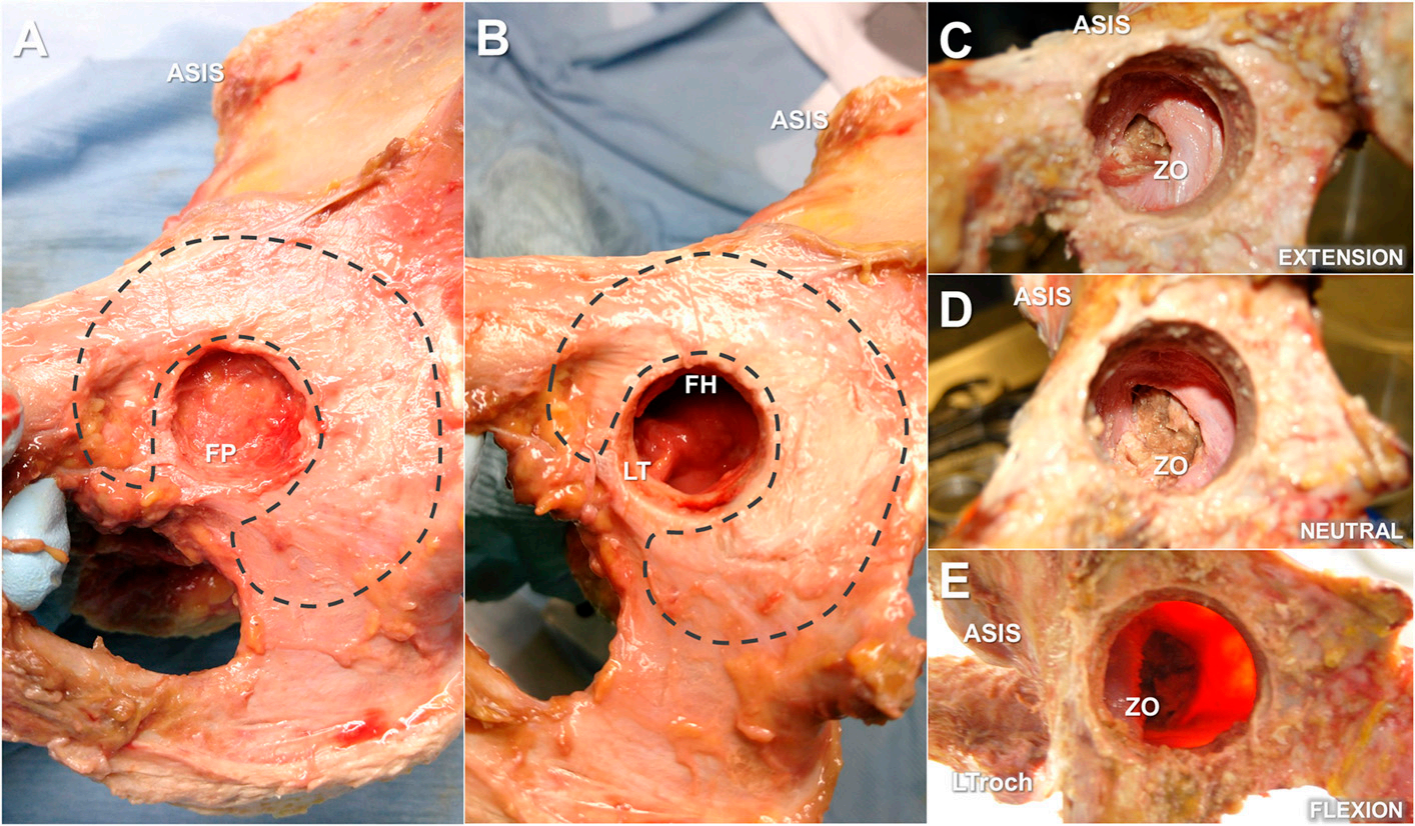
Medial-to-lateral view of a right-sided cadaveric hip specimen through progressive portal sizes and depths of the cotyloid fossa and acetabulum, indicating the small portal view of the fat pad (FP), cushioning the innervations and proprioceptors (**Fig. 2-A**); ligamentum teres (LT) tethering the femoral head (FH) at the fovea pit (with the fat pad removed) (**Fig. 2-B**); large portal view of the tightened posterior collar of the zona orbicularis (ZO) during hip extension (with ligamentum teres, acetabulum, and femoral head removed) (**Fig. 2-C**); relaxed ZO during neutral position (**Fig. 2-D**); and tightened anterior collar of the ZO during deep hip flexion (**Fig. 2-E**). The acetabular cartilage (dashed lines), anterior superior iliac spine (ASIS), and lesser trochanter (LTroch) are indicated for reference.

Human ligaments consist of predominantly type-I collagen (85%) and combinations of type III, V, VI, XI, and XIV (15%)^21,22^. Within the hip joint, higher ratios of type-III collagen in the ligamentous capsule are associated with hip instability^23,24^, whereas elevated levels in the cartilage are associated with progressive joint degeneration^25,26^. The hip joint itself is reinforced by 3 primary fibrous capsular ligaments (iliofemoral, ischiofemoral, and pubofemoral), and each serves distinct functional roles to stabilize the joint^5,8^. The iliofemoral ligament is composed of lateral (superior) and medial (inferior) fibrous branches, which insert together into the anterior inferior iliac spine of the pelvis, each extending out to attach along the femoral intertrochanteric line, forming the inverted Y-shaped ligament of Bigelow (Fig. 1), to reinforce the capsule during external rotation and extension. The ischiofemoral ligament inserts in the ischium, posteroinferior to the acetabular rim, and attaches to the posterior intertrochanteric line to reinforce the capsule during internal rotation in neutral positions as well as in combined flexion-adduction positions (i.e., FADIR [flexion, adduction, and internal rotation]). The pubofemoral ligament inserts in the superior pubic ramus and converges with the medial iliofemoral and inferior ischiofemoral ligaments to insert into the femur^7^, reinforcing the inferior capsule to restrict excessive abduction and external rotation during hip extension. Another important structure is the triangular-shaped ligamentum teres, which reinforces between the peripheral inferior acetabular notch and the fovea of the femoral head (Fig. 2). As a small auxiliary ligament overlying the fat pad, the ligamentum teres provides a conduit for small vessels and innervations to the femoral head and plays a critical role in proprioception and structural stability^27-32^, which may decrease in function with progressive age^33^. In addition to the longitudinal fibers of the primary capsular ligaments, the circular fibers of the zona orbicularis form a stability-inducing collar, which closes around the femoral neck much like an aperture mechanism. During hip extension, the posteroinferior aspect of the zona orbicularis overlaps to medialize and secure the head anteriorly, while during deep flexion, the anteroinferior aspect of the zona orbicularis medializes and secures the head posteriorly (Fig. 2). It has also been proposed that the zona orbicularis has a role in circulating synovial fluid between the central and peripheral compartments within the capsule^2^.

### Properties

Earlier uniaxial tensile studies of cadaveric hip joint specimens showed that the proximal iliofemoral ligament was larger in cross-sectional area and stiffer than the posterior capsule^1,34^. This finding emphasizes that the anterior capsule is stronger than the posterior region^35^, where perhaps the material properties of the iliofemoral ligament may be dependent on age-related changes^36^.

In addition to mechanical responses, tissue dimensions of capsular ligaments (i.e., thickness and length) can also adapt with progressive arthritis, which can further result in larger cross-sectional areas and tighter capsular ligaments^37-39^. Since the previous material characteristics were from excised capsules of cadaveric hips without pathology, more imaging modalities evolved to quantify the capsular thickness of hips with pathology using magnetic resonance imaging (MRI)^38-40^. Weidner et al., in 2012, reviewed imaging data of patients with either symptomatic cam, pincer, or mixed femoroacetabular impingement (FAI) and noted that the anterosuperior capsule (i.e., iliofemoral ligament) was the thickest region (6 mm compared with nonpathological thicknesses of 3.5 to 4.2 mm), and further noted that the zona orbicularis was more prominent in the posterior region, where the capsule was thinner and longer^39^. Interestingly, as the patients were imaged in a supine position, the thicker regions of the capsule corresponded to shorter capsule lengths, which may explain anterior hip tightness and pain. In terms of functional stability, it further suggests that the aperture mechanism of the zona orbicularis concentrically gathers these posterior thin fibers to create the stability-inducing collar during extreme hip motions. More recently, the MRI study by Rakhra et al. further compared 3 groups: hips with cam FAI, hips with chondrolabral pathology, and healthy control hips^38^. They indicated that the capsule thickness of the cam FAI group was similar to the chondrolabral pathology group. More importantly, the capsules in both pathological groups (i.e., cam FAI and diseased hips) were thicker (mean [and standard deviation], 6.8 ± 1.6 mm) than the controls (mean, 5.3 ± 2.3 mm; p = 0.026), specifically in the superior regions of the capsule. There was no correlation between alpha angle (i.e., asphericity of cam morphology) and capsule thickness. The implications of capsular dimensions and mechanical properties can help to diagnose the disease process and predict symptoms prior to degeneration. Yet, the previous studies that examined pathological cohorts did not associate capsular dimensions with stiffness^41^, which could serve as functional biomarkers of hip instability and the degenerative process. It is important to note that the imaging observations were not performed under joint-loading conditions, and earlier mechanical testing was limited to uniaxial tensile testing.

### Contributions to Joint Stability

**Fig. 3:**
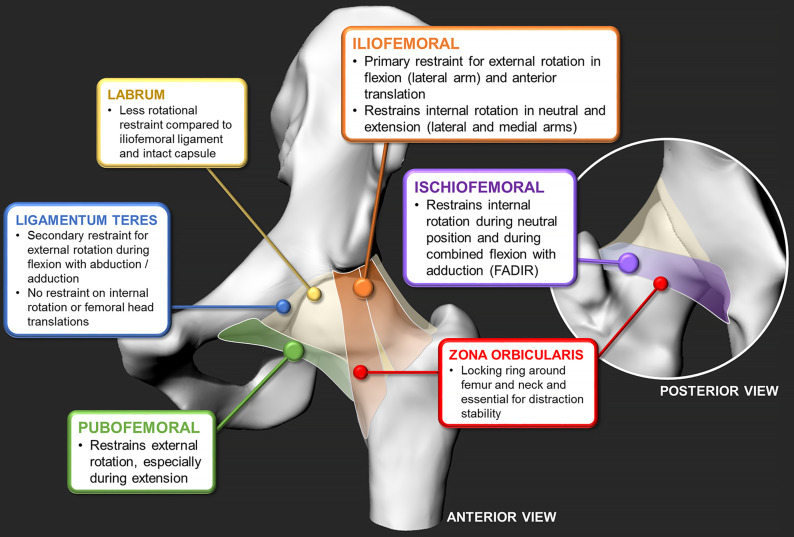
The findings of in vitro cadaveric studies on individual capsular ligament contributions to joint stability, outlining iliofemoral, ischiofemoral, and pubofemoral ligaments^4,93,44^; the zona orbicularis^45^; ligamentum teres^20,94^; and labrum^93,94^. (For a detailed summary for each study’s scope, methods, and observations, see Appendix Table I.)

To see how each capsular ligament can reinforce stability and protect the joint from edge-loading, several studies since have devised testing methods to examine the capsule within the hip joint assembly^8,30,42-44^ (Fig. 3; see Appendix Table I). The first set of tests by Martin et al. analyzed individual contributions of the capsular ligaments by performing stepwise resections^8^. The analysis provided insights into which ligament was responsible for individual rotational restraints, when release of the lateral (superior) arm of the iliofemoral ligament greatly increased external rotation in flexion and neutral positions, as well as internal rotation in extension. In a similar stepwise resection study, van Arkel et al. devised a custom joint tester and confirmed that the lateral iliofemoral ligament provided predominant external rotational restraint, whereas the pubofemoral ligament restrained external rotation and abduction during hip extension^43^. In similar studies, the labrum and the ligamentum teres acted as secondary restraints in wider external rotations^30-32,42,43^, whereas the structures of the capsule, labrum, and zona orbicularis were crucial for rotational and distraction stability^16,45^. These investigations examined hips without pathology from older donors, but were among the first laboratory studies to collectively provide important clinical insights as to which ligaments were responsible for rotational stability and which to potentially release or avoid during surgery. Finally, the role of the iliocapsularis muscle, which overlies the iliofemoral ligament, cannot be underestimated as it is an important contributor to hip stability, especially in dysplastic hips^46,47^.

## Surgical Management

### Hip Preservation Capsulotomy and Repair

**Fig. 4:**
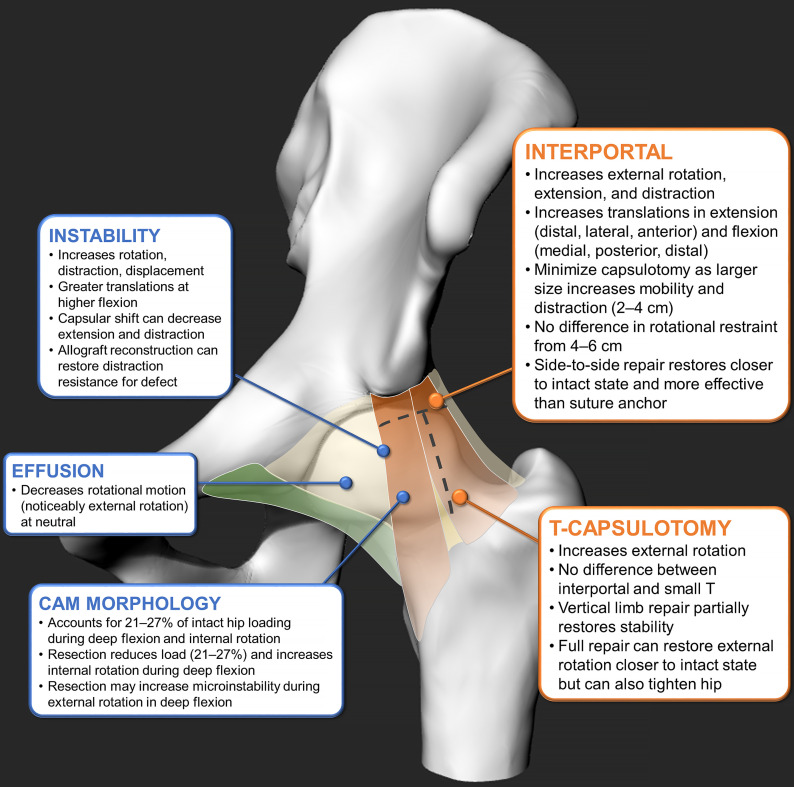
The findings of in vitro cadaveric studies on the effects of capsular conditions and surgical stages, outlining the contributions of the interportal capsulotomy^5,7,8,10,11,14,46^ and T-capsulotomy^6,11,14,15,46^ and the effects of instability^7,11-13,16,47^, effusion^9^, and cam morphology^15,16^. (For a detailed summary for each study’s scope, methods, and observations, see Appendix Tables II and III.)

With increasing laboratory evidence, the authors of several studies set off to better understand the mechanical effects of various capsulotomy types, portal sizes, repair strategies, and capsular defects on hip stability (Fig. 4; see Appendix Table II). Several in vitro studies examined conventional interportal-only^9,12,14^ and T-shaped^10,15,18^ capsulotomies, with all showing the substantial increases in motion immediately after release of the iliofemoral ligament. This ligament primarily restricts excessive hip extension and external rotation; thus, previous conventional hip arthroscopy and open surgical dislocation involve releasing the iliofemoral ligament to gain greater access to the femoral head and neck. In general, it was suggested that there were marginal differences in stability between common capsulotomy approaches (i.e., interportal and T-capsulotomy)^10,15,18^, and capsular closure restored motion to more similar intact conditions^9,10,12,14,15,18^. In studies involving similar stepwise capsular management, Philippon et al. and Baha et al. measured the differences in range of motion after each surgical stage during capsular management^15,48^. Although both studies demonstrated that common arthroscopic capsulotomy procedures (i.e., portal placement, interportal capsulotomy, and T-capsulotomy) increased the envelope of hip motion (internal-external rotation, abduction-adduction, and flexion-extension), their capsular repair and reconstruction helped to partially restore normative range of motion of the joint. In a similar stepwise capsulotomy and repair study, Weber et al. recently showed that incrementally larger interportal capsulotomies decreased distraction stability^18^, while converting a smaller interportal capsulotomy to a T-capsulotomy (i.e., an additional incision to the vertical limb) did not affect distraction resistance. Although the aforementioned studies noted substantial differences, they used hip specimens from older donors (age range, 51 to 78 years) without any hip pathologies (e.g., cam morphology and dysplasia), which may not be representative of young patients having early onset hip pain.

In terms of simulating capsular laxity and abnormalities that would warrant the need for repair or plication, Jackson et al. simulated joint instability (subjected hips were stretched in extension under 35 Nm of torque for 1 hour) and suggested that side-to-side repair only partially decreased distraction resistance, while a capsular shift approach restored joint stability^11^. Although capsular plication may help to restore joint stability in extreme cases of joint subluxation, it perhaps should not be a routine procedure for all capsular closures. More recently, Johannsen et al. examined the effects of capsular laxity by subjecting hips without pathology to anterior capsular stretching (i.e., maximal extension with external rotation), which resulted in overall joint laxity and instability^49^. They noted that the anterior capsule predominantly controlled rotation and displacement, suggesting that the iliofemoral ligaments should be carefully preserved using more conservative capsulotomies. For capsular defect cases, allograft can be a viable option to restore distraction stability^15,16^. On the other hand, Hebert et al. compared the effects of hip effusion (simulated with an injection of 10 mL of saline solution) and capsular tears^13^. They noticed that effusions associated with arthritic conditions decreased hip mobility, while a capsular tear naturally increased instability. Therefore, as many routine surgical hip preservation and arthroplasty procedures seek to restore joint function in the prearthritic or already arthritic hip, it is crucial to consider intracapsular conditions, how inherently tight the hip in question is (i.e., joint effusion and level of degeneration or stiffness)^50^, or if the hip is too inherently unstable to warrant plication. As the specimens were from donors who were slightly older in age, it may not be appropriate to interpret the mechanical properties without considering age-related changes in the musculoskeletal system^36,51^. These results from in vitro cadaveric studies should be interpreted with caution, especially if the demographics and pathological groups were not appropriately represented in the experiments (i.e., healthy hips with no pathology were examined instead of those with osseous deformities or pathologies).

It is essential to understand that capsular characteristics and mechanical properties of the hip with pathological conditions are different (i.e., a thicker, stiffer capsule) than a healthy joint^38,50,52^; thus, the need for full capsular closure may depend on several other confounding factors (e.g., age, sex, osseous anatomy, and muscle function)^53-55^. More importantly, since unrepaired capsulotomies have been shown to heal within 24 weeks postoperatively^56^, and completely resecting the iliofemoral ligament does not destabilize the native hip^43^, there is evidence that not all capsulotomies need to be repaired after arthroscopy, especially when capsular contracture may be part of the pathological process. It is still unclear what leads to inherent or iatrogenic instability; thus, if the native head size is not substantially reduced or altered, capsular repair in the setting of a small arthroscopic capsulotomy may not be necessary in the otherwise congruent and stable hip^4,54,56^. Recently, a periportal capsulotomy technique (i.e., midanterior and anterolateral portal dilations) showed that it can preserve the iliofemoral ligament, without necessitating capsular closure^57,58^; however, it is unclear if this applies to dysplastic hips^59^.

### Effects of Hip Preservation Surgery

Although the functional anatomy and biomechanics of the hip joint and encapsulating ligaments have been well described, surgical intervention and capsular management in hip arthroscopy and arthroplasty continue to raise many pressing concerns. With emerging interest in improving capsular management for hip preservation and postoperative joint arthroplasty mechanics, it is imperative to delineate the contribution of each surgical stage toward functional mobility and stability. The following recent in vitro laboratory studies contributed substantially to the understanding of hip arthroscopy and arthroplasty surgery, addressing several issues of demographics, stepwise surgical stages, varying implant parameters (e.g., implant type, head size, neck length, and anterior or posterior approach), and provided detailed insights into capsular function, mechanics, and management (see Appendix Table III).

**Fig. 5:**
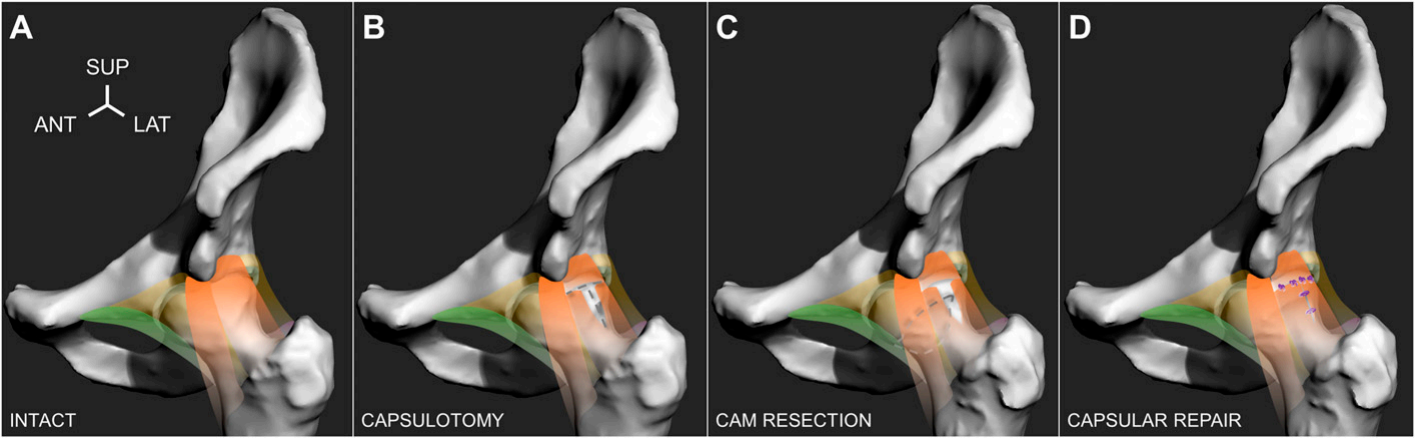
Four surgical stages depicted on a left-sided hip, which included the intact hip with cam morphology, were tested (with orange indicating the iliofemoral ligament; green, the pubofemoral ligament; and yellow, the encapsulating tissue) (**Fig. 5-A**); capsulotomy, in which the iliofemoral ligament was incised to create a T-capsulotomy (dashed lines) (**Fig. 5-B**); cam resection, in which the cam morphology was resected through the incised capsule (dashed lines) (**Fig. 5-C**); and capsular repair, in which the incised portal was closed using simple, interrupted sutures (purple knots) (**Fig. 5-D**). ANT = anterior, LAT = lateral, and SUP = superior. (Adapted, under CC BY 4.0 [https://creativecommons.org/licenses/by/4.0/], from: Ng KCG, El Daou H, Bankes MJK, Rodriguez Y Baena F, Jeffers JRT. Hip joint torsional loading before and after cam femoroacetabular impingement surgery. Am J Sports Med. 2019 Feb;47[2]:420-30. Published by SAGE Publishing. https://doi.org/10.1177/0363546518815159.)

**Fig. 6:**
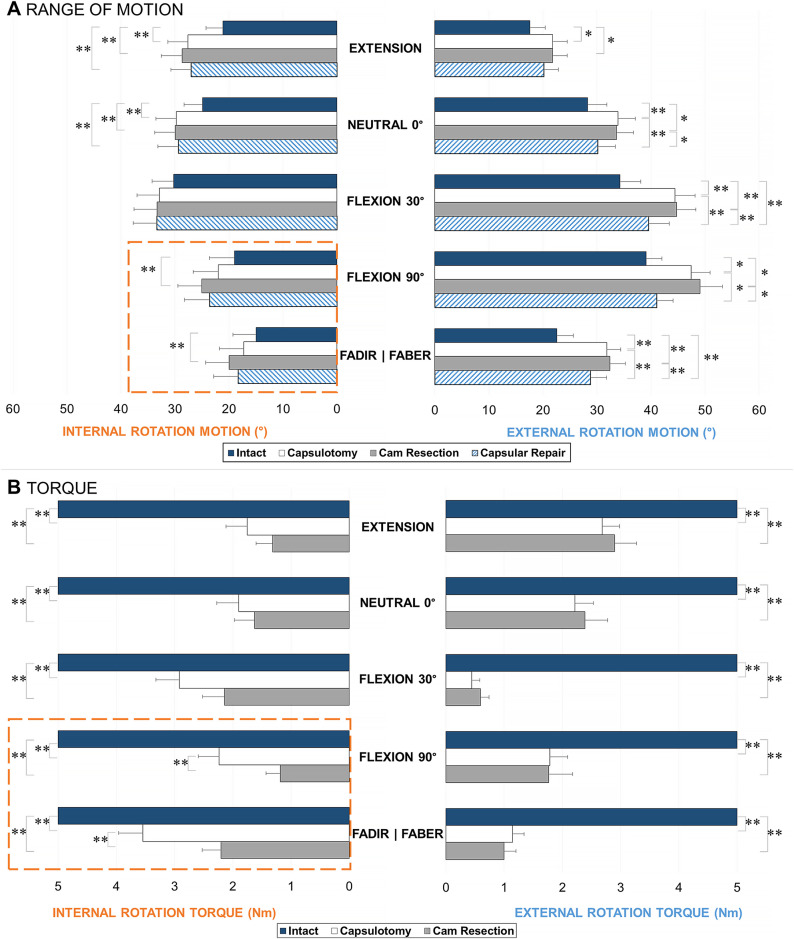
Range of motion (**Fig. 6**-**A**) and torque loading (**Fig. 6-B**) in internal and external rotation, at each stage of testing: intact, capsulotomy, cam resection, and capsular repair (range of motion only), reported as the mean and standard error. Cam resection further increased internal rotation in the deep flexion positions (highlighted dashed lines). *The difference is significant (p < 0.05). **The difference is significant (p < 0.01.) FADIR = flexion-adduction and internal rotation, and FABER = flexion-abduction and external rotation. (Adapted, under CC BY 4.0 [https://creativecommons.org/licenses/by/4.0/], from: Ng KCG, El Daou H, Bankes MJK, Rodriguez Y Baena F, Jeffers JRT. Hip joint torsional loading before and after cam femoroacetabular impingement surgery. Am J Sports Med. 2019 Feb;47[2]:420-30. Published by SAGE Publishing. https://doi.org/10.1177/0363546518815159.)

Although it has been well established that surgically resecting the cam morphology can delay the pathomechanical process of subchondral bone stiffening^60,61^, cartilage degeneration^62,63^, and adverse loading^64,65^, it was elusive how much the actual morphology or any of the individual surgical stages contributed to these aspects. In what we believe is the only documented in vitro cadaveric study that has examined a well-represented pathological cohort with cam morphologies, Ng et al. evaluated a large group of hip specimens with moderate-to-high alpha angles (representative of the symptomatic cam population) from young, male donors^19^. The study examined how the capsulotomy, cam resection, and capsular repair affected passive range of motion and torque loading (Fig. 5), using a 6-degrees-of-freedom robotic testing platform^66^. The study quantified the contribution of the cam deformity, which accounted for 21% to 27% of the torsional loading of the intact hip in deep flexion and internal rotation (Fig. 6), where the specimens indicated secondary at-risk parameters of smaller femoral neck-shaft^67-71^ and higher spinopelvic incidence angles^72-74^. Capsular repairs restrained external rotation during deep flexion and were ineffective in other testing positions, partly because of the inherently large native head that maintained joint stability and partly because the cam deformities were not overresected (i.e., no proximal concavity or “cookie bite”). As the femoral head is naturally conchoidal^75^ and may be precisely shaped to maintain the labral seal and effectively distribute load^76^, there may be suboptimal outcomes if a smaller cam morphology is resected. Translations of the hip joint center should be further examined to help characterize microinstability over the series of surgical stages^17,20,49^. In addition, the balance between capsular repair and plication should be compared with established objective measures to minimize iatrogenic instability in hips with pathology^6,19^.

### Effects of Hip Arthroplasty Surgery

**Fig. 7:**
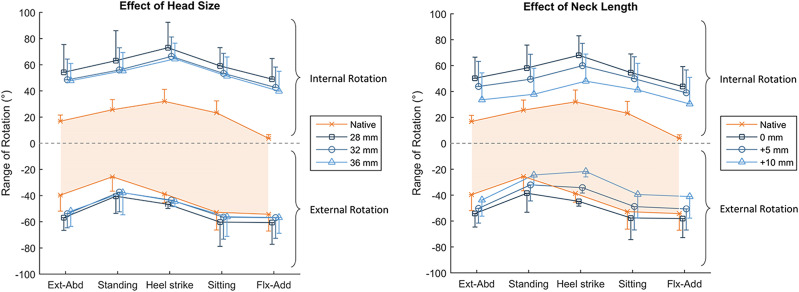
After total hip arthroplasty, the effects of head size (left), with anatomical neck length, and increasing neck lengths (right) on rotational range of motion are depicted. The x axis denotes the testing positions, and the y axis denotes the internal-external rotation (positive-negative). The shaded orange area dictates the measured range of native hip rotation. Increasing head size had little effect, whereas lengthening the neck tightened the hip capsule and reduced external rotation in some hip positions. The values are given as the mean, and the error bars indicate the standard deviation. Ext-Abd = extension and abduction, and Flx-Add = flexion and adduction. (Reproduced from: van Arkel RJ, Ng KCG, Muirhead-Allwood SK, Jeffers JRT. Capsular ligament function after total hip arthroplasty. J Bone Joint Surg Am. 2018 Jul 18;100[14]:e94.)

In an effort to characterize capsular function after total hip arthroplasty, van Arkel et al. inserted the femoral head, neck, and cup implants through a cotyloid fossa portal and, by doing so, preserved the entire intact joint capsule^77^. The authors reported that the smaller head sizes removed the natural tensioning ability of the capsule to restrain rotational motion (Fig. 7). The anterior capsule was less affected, particularly in flexion, as it had less of a dependence on ligament wrapping compared with the posterior ischiofemoral ligament. This further suggests that an intact zona orbicularis, which is more prominent in the posterior region^39^, can provide more help to medialize and secure the hip during extreme flexion-extension (without abduction-adduction and internal-external rotations) than the primary 3 ligaments. More importantly, a shorter neck length increased hypermobility, whereas a longer neck overtightened the anterior capsule (Fig. 7). These findings highlight the critical interaction between optimizing hip biomechanics and gait mechanics^78-81^.

**Fig. 8:**
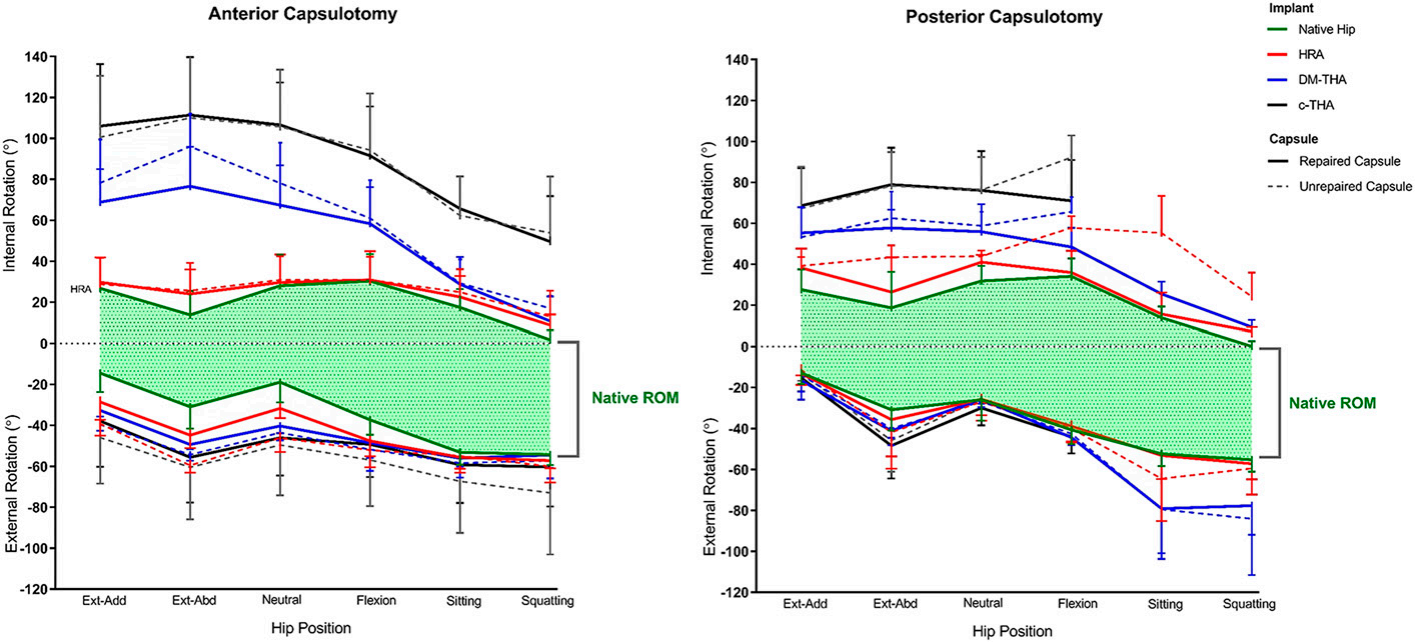
The effects of the anterior (left) and posterior approaches (right) are examined with various types of implants: hip resurfacing arthroplasty (HRA; red), dual mobility arthroplasty (DM-THA; blue), and conventional total hip arthroplasty (c-THA; black) after unrepaired (dashed) and repaired conditions (solid). The x axis denotes the testing positions, and the y axis denotes the internal-external rotation (positive-negative). The shaded green area dictates the measured range of native hip rotation. The larger head size of the hip resurfacing arthroplasty demonstrated a range of motion that was more similar to the intact condition. The posterior approach showed higher dislocation risks during deep flexion. The values are given as the mean, and the error bars indicate the standard deviation. Ext-Abd = extension and abduction, Flx-Add = flexion and adduction, and ROM = range of motion. (Reproduced, with permission, from: Logishetty K, van Arkel RJ, Ng KCG, Muirhead-Allwood SK, Cobb JP, Jeffers JRT. Hip capsule biomechanics after arthroplasty: the effect of implant, approach, and surgical repair. Bone Joint J. 2019 Apr;101-B(4):426-34. ©2019 The British Editorial Society of Bone & Joint Surgery).

Logishetty et al., using paired hip specimens (n = 16; 8 left and 8 right), compared conventional hip arthroplasty with dual-mobility total hip arthroplasty and hip resurfacing arthroplasty^82^. The left-sided hips all underwent the direct anterior approach and capsulotomy, while the right-sided hips underwent the posterior surgical approach and capsulotomy. Because hip resurfacing arthroplasty preserved much of the near-native head size and femoral neck, it enabled a range of motion with marginal increases in hypermobility compared with the intact stage. Dual-mobility arthroplasty slightly increased range of motion, while total hip arthroplasty further increased hypermobility and lost most of the inherent capsular function. Although the posterior approach and capsular repair provided more normal function during low hip flexion and extension positions, with a shortened capsule after repair, it was evident that the total hip arthroplasty through the posterior approach demonstrated a higher likelihood of instability during deep flexion (Fig. 8). In contrast, the anterior approach functioned better in deep hip flexion and was not a dislocation risk after total hip arthroplasty. This was attributed not only to the preserved posterior ischiofemoral ligament but also perhaps to the preserved zona orbicularis, which helped to maintain distraction stability^45^ and highlighted the role of the obturator externus, which can pull taut along the same line as the posterior capsule^83,84^. That study illustrated the complex interaction between native hip structures in the context of hip arthroplasty (i.e., capsule, ligaments, and muscles) and joint mechanics specific to a surgical approach^82^. Regardless of approach, the near-native head size of a hip resurfacing arthroplasty and subsequent capsular repair preserved postoperative joint function.

## Overview

Recent research has focused on hip mechanics and our understanding of capsular function after hip preservation and arthroplasty, further highlighting fundamental capsular characteristics in the context of instability as well as optimizing hip function. Most of the work was conducted in in vitro cadaveric studies, which examined material characteristics of capsular ligaments, functional joint mobility and stability, or surgical management techniques. Little in vivo work in human subjects has been published.

In joint preservation surgery, there are ongoing debates as to whether to repair the capsule^6,10,54,56,85-87^. The decision to repair the capsule should not be a simple yes-or-no choice, as there should not be a general one-size-fits-all solution. Although the unrepaired capsule has been associated with inferior outcomes^6,86^, several confounding factors should still be considered^87,88^, such as the size and location of the capsulotomy, osseous morphology, and degree of surgical correction as well as native joint mobility. The effects of these factors are evident in the many cases of iatrogenic instability arising in patients with milder deformities who underwent overcorrection, resulting in ineffective labral seals and load distributions^76,89-91^, which were further compounded by a deficient capsule^92^. As capsular repair can substantially decrease external rotation during deep hip flexion, complete capsular repair or augmentation may be most effective for larger capsulotomies or overresected cams^18,85^; however, it should be cautiously performed to avoid overtightening the joint^19^.

Since surgical management of FAI and other common hip pathologies in young adults can be effective in minimizing the risks of adverse cartilage loading and degeneration^19,61,64,65,70^, more research needs to be done to examine where changes occur first (i.e., subchondral bone, cartilage, or capsule) in response to varying joint morphology and mechanics. This is certainly the case for the zona orbicularis. In both arthroscopic and arthroplasty cadaveric studies^54,82^, the hip joint stability was negatively affected when the vertical limb of the anterior capsulotomy extended past the intertrochanteric line and with a conventional posterior approach. The zona orbicularis is not only important for resisting joint distraction but also for the locking effect of the stability-inducing collar, which seems to play a role in preventing edge-loading (Video 1). Finally, a better understanding of capsular properties and hip biomechanics provides further insight into how implant type (e.g., resurfacing, dual-mobility, and conventional arthroplasty), reconstruction parameters (e.g., head size and offset), and surgical approaches (e.g., anterior or posterior) can impact hip range of motion and stability as well as the critical contribution of the overlying hip musculature.

**Video 1:** To view the internal structures of the capsule, the acetabulum and femoral head were removed from the medial cotyloid fossa portal. Similar to the aperture of a camera, the zona orbicularis closes.

## Appendix

Supporting material provided by the authors is posted with the online version of this article as a data supplement at jbjs.org (http://links.lww.com/JBJS/F512).
